# Impact of Nitrite Supplementation on Bioactive Peptides during Sausage Processing

**DOI:** 10.3390/foods12020407

**Published:** 2023-01-14

**Authors:** Rongyu Zang, Qunli Yu, Zonglin Guo

**Affiliations:** College of Food Science and Engineering, Gansu Agricultural University, Lanzhou 730070, China

**Keywords:** sausage, nitrite, antioxidant polypeptide

## Abstract

The goal of this investigation was to examine the impact of nitrite supplementation on the concentration, antioxidant properties, and species of antioxidant peptides in fermented sausages. The polypeptide concentration in nitrite-supplemented sausages was markedly elevated during sausage processing compared to the blank control (*p* < 0.05). Moreover, nitrite supplementation in fermented sausages markedly enhanced the DPPH, as well as the ABTS, hydroxyl radical, and superoxide anion free radical scavenging abilities (FRSA) of polypeptides (*p* < 0.05). The ferrous ion chelating ability was also significantly enhanced (*p* < 0.05). Based on the liquid chromatograph-mass spectrometer (LC-MS) analysis of the sausage, LPGGGHGDL, TKYRVP, FLKMN, SAGNPN, GLAGA, LPGGGT, DLEE, GKFNV, GLAGA, AEEEYPDL, HCNKKYRSEM, TSNRYHSYPWG, and other polypeptides exhibited antioxidant properties. Moreover, the number of species of antioxidant polypeptides in the nitrite-supplemented sausage was greater in comparison to the controls. Based on this evidence, it may be concluded that nitrite supplementation positively modulated antioxidant polypeptide formation in fermented sausages, thereby providing strong evidence that nitrite supplementation significantly enhances sausage quality.

## 1. Introduction

Fermented sausage is prepared with microbial fermentation and involves the drying and maturation of minced meat combined with animal fat, salt, sugar, starter, and a variety of spices [1]. Due to their unique color, texture, and flavor characteristics, fermented sausages are highly preferred by Chinese consumers, and are among the most popular fermented meat products [2]. 

Nitrate and nitrite usage in the meat industry is strictly regulated due to their toxicity and carcinogenicity [3]. Many recent studies have, therefore, developed alternatives to nitrites. Based on research by Xin et al. [4], lemon seed essential oil (LSO) and pitaya peel (PP) extracts were able to successfully preserve mutton and minimize nitrite usage during the curing process. Based on their data, the nitrite-free sausages appear less red and are darker in comparison to the nitrite-treated products. However, this study did not utilize a large variety of prokaryotics. Instead, the researchers mostly employed Staphylococcus xylosus and Lactobacillus sakei, and the chemical/physical parameters were also not significantly varied. Despite their many disadvantages, nitrates and nitrites are still frequently used in the meat industry, particularly during meat curation and fermentation [5,6]. Their usage delays oxidative rancidity and establishes the classic flavor and color associated with traditional bacon [7]. Nitrite is widely used in cured meat products. It aids in fixation, is bacteriostatic, and reduces meat lipid peroxidation when added in appropriate quantities [8]. Excess nitrite addition can convert nitrite to N-nitrosamines (N-NA), ultimately leading to nitrite poisoning [9]. Therefore, an increasing number of investigations are currently pursuing useful alternatives to nitrite. Unfortunately, owing to the high cost of replacement, nitrite remains irreplaceable in meat processing in actual production lines [10]. Relative to nitrate, which forms nitrosamines in high concentrations and causes undesirable reactions, nitrite has the overwhelming advantages of antioxidation, color development, and bacteriostatic activity [11]. Nitrite interacts with NO and heme iron/Mb to suppress oxidation in cured meat [12]. The antioxidant property of MbFeIINO and, potentially, other nitrite-based compounds largely prevents oxidative rancidity in nitrite-preserved meat [13]. Until now, limited investigations have examined the antioxidant mechanisms of NaNO_2_ in meat systems. In the carotene-linoleic acid model system, MbFeIINO strongly suppressed oxygen consumption in a lipid peroxidation model [14]. Moreover, methyl linoleate usage as an NO substrate induces high-iron myoglobin free radicals to participate in oxidative stress inactivation [15].

During sausage fermentation, endo- and exopeptidases present within meat break meat proteins down into protein fragments and polypeptides [16,17]. In the early stages of sausage fermentation, proteolysis is initiated by endopeptidases such as calpains and cathepsins, particularly cathepsins B, H, and L, within the first few weeks of processing. This results in the degradation of protein to protein fragments and peptides, which are then further hydrolyzed via exopeptidases into smaller free amino acids and peptides [18]. It is generally understood that the presence of these products imparts a unique flavor to mature sausages [17]. Moreover, the generation of massive amounts of peptides during ham processing potentially benefits body function and consumer health. Owing to the presence of certain amino acid residues, the aforementioned peptides can cross the digestive epithelial barrier and enter the circulatory system. This property enables these peptides to reach distant organs and cause a positive impact. As such, the formation of free amino acids and peptides is considered essential for the good organoleptic properties and nutritional value of sausages [19]. The protein hydrolysis status, as well as the number and type of bioactive peptides produced during sausage fermentation, are generally limited by several factors, such as the type of raw material, type and activity of muscle enzymes, type and quantity of excipients, processing conditions, and processing duration [18,20,21]. A thorough understanding of the association between protein hydrolysis and processing parameters will undoubtedly enhance the optimization of sausage processing during the manufacturing processes [19]. However, the effect of nitrite on proteolysis during sausage fermentation has not yet been investigated, as it is a substance that must be introduced during the current processing of fermented sausage. This study primarily focused on examining the effects of nitrite supplementation during sausage fermentation, particularly in terms of sausage quality. Additionally, the types and antioxidative properties of the formed polypeptides were investigated. To accomplish this, the peptide concentration, antioxidant properties, and species production during sausage fermentation were assessed at various processing times (0, 4, 18, 30, and 55 days post-processing). Our findings will surely advance the science of sausage fermentation and add to the body of knowledge currently available regarding quality control.

## 2. Materials and Methods

### 2.1. Materials

Yellow cattle (about 400 kg/head of live cattle) from the Gannan Kangmei Animal Husbandry and Food Co., Ltd., Gansu, China were selected and slaughtered based on the GB/t1947-2018 standard. Spices were acquired from the Taohai Market, Lanzhou City, Gansu Province. Chemical reagents such as DPPH, ABTS, and alcohol were acquired from Merck (Darmstadt, Germany). All chemicals utilized were of analytical grade. Salt was obtained from the China National Salt Industry Group Co., Ltd., Gansu, China. White granulated sugar was purchased from Nanning Sugar Co., Ltd., Guangxi, China. Chicken essence was acquired from Shanghai Totole Food Co., Ltd., Shanghai, China, and monosodium glutamate was procured from Henan Lotus Co., Ltd., Henan, China. All aforementioned spices and seasonings were food-grade.

### 2.2. Preparation of Sausage

The sausage production process as well as the flavoring type and weight were in complete accordance with the factory requirements. Meat from the hind legs of yellow cattle was ground in a meat grinder (LZ223a, Supor Co., Ltd., Zhejiang, China) for 5 min. Subsequently, seasoning and spices (salt (21.5 g/kg), white sugar (25.0 g/kg), sodium ascorbate (0.5 g/kg), and LPH (0.215 g/kg) were introduced and mixed well. Sodium nitrite (0.15 g/kg) was introduced to the treated meat, and not to the controls. The meat was then poured into a natural casing and placed in a LHS-150SC constant temperature and humidity box (Yiheng Technology Co., Ltd., Shanghai, China) for fermentation at 37 ± 2 °C and 50 ± 2% relative humidity. Six sausage slices were then extracted from various hams *(n* = 6) at specified processing durations, namely 0, 4, 18, 30, and 55 d, respectively. All analyses were performed in triplicate.

### 2.3. Physicochemical Analysis of Sausage

Lipid oxidation was assessed via a slightly modified thiobarbituric acid reactive substance (TBARS) procedure. Briefly, 5 g of minced BF sample underwent homogenization in 20 mL trichloroacetic acid (TCA) (5%, *m*/*v*) and 500 µL butylated hydroxytoluene (BHT) (4% in ethanol) prior to centrifugation at 12,000 rpm for 10 min at 4 °C. Subsequently, 4 mL of the supernatant was vortexed thoroughly in 4 mL 0.02 M thiobarbituric acid (TBA), followed by successive incubation in a boiling water bath (JP Selecta, Barcelona, Spain) for 60 min to establish color, and another incubation in ice water for 15 min before analysis. The resulting supernatant was assessed at a 532 nm wavelength via an ultraviolet–visible (UV–Vis) spectrophotometer (Cary 60; Agilent Technologies, Santa Clara, CA, USA) against a blank containing 4 mL TCA and 4 mL TBA. The TBARS quantity was then computed from the standard calibration curve using 1,1,3,3-tetraethoxypropane (TEP) (0.1–2 μg/mL malondialdehyde (MDA)) as the standard, and then expressed as mg MDA/kg BF muscle sample (wet weight basis).

Protein oxidation was assessed via the carbonyl index of BF samples, as evidenced by the 2,4-dinitrophenylhydrazine (DNPH) derivatization analysis. The protein carbonyl count was determined at 370 nm using a UV–Vis spectrophotometer (Cary 60; Agilent Technologies, Santa Clara, CA). In the meantime, the final pellets were rinsed and re-suspended in 20 mM sodium phosphate buffer at pH 6.5 with 6 M guanidine hydrochloride, and were subsequently processed for absorbance measurement at 280 nm. The purpose of this step was to compute protein concentration via an absorption value against a calibration curve plot, using bovine serum albumin (BSA) as a standard (0.2–2 mg/mL). The final carbonyl index result was presented as mM hydrazones/mg protein using the absorption coefficient of the protein hydrazones (22.0 mM^–1^ cm^−1^).

The sensory quality of the cooked pork sausages was then evaluated using ranking tests (ISO 8589, 2007). For this purpose, 50 students and teachers were recruited from Food Science and Technology at the Gansu Agricultural University of China (25 males and 25 females, 25–50 years old). Sausages were incubated at room temperature (RT) for 15 min before serving. They were then sliced into 2 mm thick pieces and presented on a white porcelain plate, labeled using a three-digit sample number. The meat evaluators were asked to classify the samples in numerical order based on sensory qualities, such as color and flavor, according to their personal preferences (from extreme dislike, 1, to extreme like, 7).

### 2.4. Extraction of the Crude Peptide from Sausage

Using a DY89-IIhomogenizer (Xinzhi Machinery Co., Ltd., Ningbo, China), the samples were crushed in 25 mL acetone at 10,000 rpm for 3 min, followed by slow stirring at RT for 15 min and centrifugation at 4000× *g* and 4 °C for 30 min in a KL04A centrifuge (Kaida Scientific Instrument Co., Ltd., Hunan, China). The resulting pellet was dried overnight (ON) at 25 °C in an oven.

The dried sample (5 g) was homogenized in 0.01 mM HCl solution for 8 min, followed by centrifugation at 12,000× *g* for 20 min at 4 °C. The resulting supernatant underwent filtration via filter paper (0.45 nm). Next, to eliminate protein components, three times as much ethanol was introduced to the filtrate prior to incubation at 4 °C for 12 h and subsequent centrifugation at 12,000× *g* for 5 min at 4 °C, after which the supernatant was collected. The supernatant was then centrifuged in a 3 Kda ultrafiltration tube, and the filtrate was collected and freeze-dried for future analysis.

### 2.5. Determination of Peptide Concentration from Sausage

The o-phthalaldehyde method (OPA) was employed for the determination of the extracted peptide concentration. The OPA mixture comprised 25 mL of 100 mM sodium tetraborate, 2.5 mL of 20% (*m*/*v*) sodium dodecyl sulfate (SDS), 40 mg of OPA in 1 mL methanol, 100 μL of β- mercaptoethanol, and 21.4 mL of distilled water (DW). To initiate the process, 50 μL of the sample was introduced to 2 mL of the OPA mixture. Following a 4 min reaction at RT, absorbance was determined at 340 nm. Initially, 5 mL of the enzymatic hydrolysate was combined with an equal volume of 15% TCA solution. Following vigorous shaking, the mixture was incubated at RT for 30 min prior to centrifugation at 5000 rpm for 15 min at 4 °C. The supernatant absorbance was measured at 540 nm using the biuret method. Lastly, the soluble peptide content was determined using the standard curve (y = 0.0459x − 0.0003 R^2^ = 0.9998). 

### 2.6. Determination of the Antioxidative Capacity of Peptides in Sausage

#### 2.6.1. Diphenyl-2-picrylhydrazyl (DPPH) Free Radical Scavenging Ability (FRSA)

In both nitrite-treated and untreated fermented beef sausages, the polypeptide DPPH FRSA was assessed [22]. To accomplish this, 50 μL of the sample, including whole extracts and peptide fractions, 250 μL of ethanol (100%), and 125 μL of DPPH solution (0.02% in ethanol) were combined and incubated at RT without light for 60 min, prior to absorbance measurement at 517 nm. A low value indicated an enhanced FRSA within samples. The background as well as the negative and positive control samples were also prepared as mentioned above, except that the sample was substituted for the same solvent that was used to re-suspend the entire extracts, peptide fractions, DW, and GSH. Each sample was replicated three times. The FRSA of DPPH was computed as follows:

$$\mathrm{DPPH}\;\mathrm{FRSA}\;\left(\%\right)=\frac{A_{blank}-A_{sample}}{A_{blank}-A_{background}}\times 100\%$$

where *A_sample_*, *A_background_*, and *A_blank_* denoted absorbances of the sample, background, and blank controls (without peptides), respectively.

#### 2.6.2. Azino-bis(3-ethylbenzothiazoline-6-sulfonic acid) (ABTS) FRSA

This assay followed the protocol of Re et al. [23]. In short, the sausage polypeptides were prepared in 0.1 mg/mL concentration of varying masses with DW. Then, 0.2 mL of 7.4 mmol/L ABTS and 0.2 mL of 2.6 mmol/L K_2_S_2_O_8_ were combined and incubated at RT without light for 15 h. The phosphate buffer solution (pH 7.4) was used to dilute the mixed static reagent 50 times to serve as the ABTS working solution. Subsequently, a 0.2 mL volume fraction of 95% ethanol solution was mixed with 0.8 mL ABTS solution, and then stirred for 6 min prior to absorbance measurement at 734 mm. All experiments were performed in triplicate, and the resulting data were statistically assessed.

#### 2.6.3. Hydroxyl FRSA

Using a modified form of a previously published protocol (Vanvi and Tsopmo, 2016), the hydroxyl FRSA was assessed. To prepare for the assay, varying mass concentrations of 0.1 mg/mL sausage polypeptides were created in DW. Next, 1.0 mL of 9.0 mmol/L FeSO_4_ 7H_2_O solution, 1.0 mL of 9.0 mmol/L salicylic acid-ethanol, and 1 mL of loach protein polypeptide were mixed, and 1 mL of 8.8 mmol/L H_2_O_2_ was introduced prior to heating the solution in a water bath at 37 °C for 0.5 h. The absorbance was measured at 510 nm. All experiments were conducted thrice, and the resulting data were assessed statistically.

#### 2.6.4. Superoxide Anion ($O_{2}^{.-}$) FRSA

The protocol by Alrahmany and Tsopmo [24] was employed for this assay. Briefly, each hydrolysate (80 µL) was combined with 80 µL of 50 mM Tris–HCl buffer in a clear round-bottom 96-well microplate in the dark. Nex t, 70 µL of pyrogallol (1.5 mM) in 10 mM HCl was introduced to the samples. The absorbance was measured at 420 nm every 20 s for 4 min at RT. Three repeated measurements of a sample were taken at a one-time point. The superoxide anion FRSA was computed as follows:

$$O_{2}^{.-}\;activity\;\left(\%\right)=\frac{\left(△A_{control}/min\right)-\left(△A_{sample}/min\right)}{\left(△A_{control}/min\right)}$$

### 2.7. Iron-(II) Chelating Assay

This assay was conducted as described previously [25], with several modifications. Briefly, 50 μL sausage polypeptide solutions were prepared at varying fermentation stages to a mass concentration of 0.1 mg/mL. Individual solutions were combined with 185 μL methanol and 5 μL FeCl_2_ solution (2 mmol/L), and the reaction was initiated with 10 μL phenanthrozine (5 mmol/L). Following a 10 min incubation at RT, the absorbance *A_1_* was recorded at 562 nm. In the control samples, the ferrozine solution was substituted with ultrapure water, and absorbance was determined as *A_2_*. Ethylene diamine tetraacetic acid served as the positive control, and its absorbance was recorded as *A_0_*. All samples were prepared together simultaneously, and three repeated measurements of absorbance were taken. The ferrous ion chelation rate was computed as follows:

$$\mathrm{Iron}\;\left(\mathrm{II}\right)\mathrm{chelation}\;\left(\%\right)=\left(1-\frac{A_{1}-A_{2}}{A_{0}}\right)\times 100\%$$

### 2.8. LC-MS Mass Spectrometry

Sausage peptides at varying stages of fermentation underwent separation via EASY-nLC1000 nanoscale liquid chromatography and a Q-Exactive mass spectrometer. The loading and analytical columns were Thermo scientific EASY columns 2 cm × 100 μm, 5 μm-C18, and 75 μm × 100 mm, 3 μm-C18, respectively, with their corresponding temperatures adjusted to 40 °C. Dried peptides were dissolved in a mixture comprising 2% (*v*/*v*) acetonitrile/0.1% (*v*/*v*) formic acid prior to insertion into the LC-MS system. The mobile phases were 100% water/0.1% formic acid (buffer A) and 100% (*v*/*v*) acetonitrile/0.1% (*v*/*v*) formic acid (buffer B). Column equilibration was carried out using 95% liquid A. Sample loading was achieved via an autosampler, and separation was carried out via the analytical column. The liquid phase gradients were as follows: 0–50 min, liquid B linear gradient ranging from 0–35%; 50 min–55 min, liquid B linear gradient ranging from 35–100%; 55 min–60 min, 100% liquid B. The hydrolysis products subsequently underwent desalting and separation via high-performance liquid chromatography prior to analysis via a Q-Exactive mass spectrometer. The analysis had a duration of 60 min and was conducted in positive ion detection mode. The precursor ion scanning range was between 300–1800 *m*/*z*, the primary mass spectrometer resolution at 200 *m*/*z* was 70,000, the AGC target was 3e6, and the primary ion maximum IT was 50 ms. The polypeptides and polypeptide fragments’ mass-to-charge ratios were determined. Following individual full scans, 20 fragment spectra (MS2 scan) were acquired. The MS2 resolution was adjusted to 200 *m*/*z*, with a 17,500 rate. The Microscan was 1, the isolation window was 2 *m*/*z*, the secondary maximum IT was 60 ms, the MS2 activation form was HCD, the collision energy was 27 eV, the dynamic exclusion was 60 s, and the normalized underfill ratio was 0.1%. Subsequently, the MaxQuant software was employed to screen the corresponding database in order to obtain results pertaining to the identified protein. The UniProt Bovine fasta (total sequence: 32,200, download time: 21 September 2021) was selected as the screening database. The library screening parameters were adjusted as follows: enzyme: trypsin; missed cleavage sites: 2; fixed modification: carbamidomethyl (C); dynamic modification: oxidation (M) and acetyl (protein N-term). The identified proteins had FDR < 0.0l.

### 2.9. Data Analysis

Data were assessed using analysis of variance (ANOVA) and Duncan’s multiple range test at 5% significance (SPSS 17.0). The results are expressed as mean ± standard deviation (SD).

## 3. Results and Discussion

### 3.1. Effect of Nitrite Addition Physicochemical Properties

**Figure 1:**
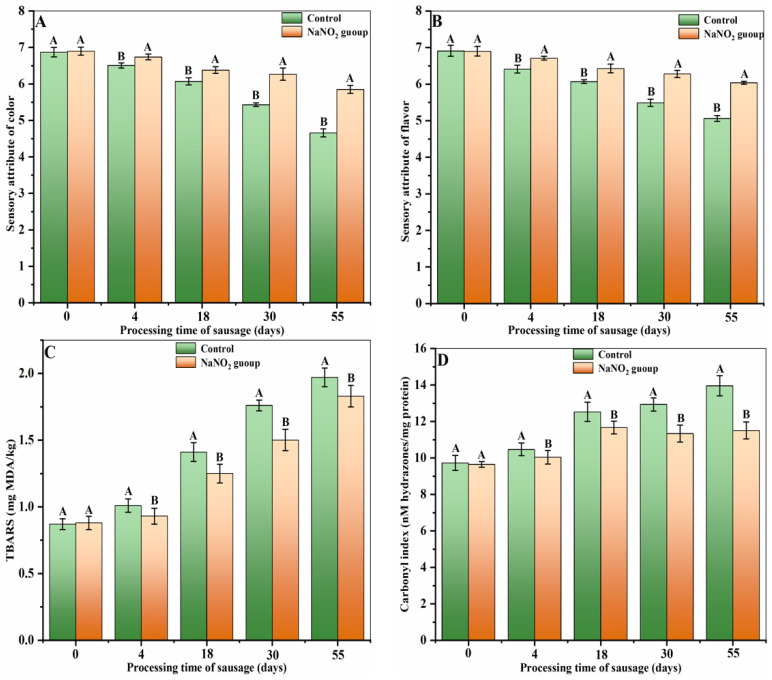
Dynamic color changes (**A**), flavor (**B**), TBARS (**C**), and carbonyl index (**D**). Different uppercase letters in a column indicate significant differences among treatments during storage time (days) for each treatment (*p* < 0.05).

Alterations to sensory attributes, such as the color and flavor of both the control and treated samples, are provided in Figure 1A,B, respectively. The color and flavor property scores decreased with the increase in processing time. During prolonged processing durations, lipid oxidation, as evidenced by the TBARS value, was markedly elevated (*p* < 0.05) (Figure 1C). This was likely due to the pro-oxidative activity of the metallic ions present within the salt used in the curing process. Furthermore, based on Figure 1D, the carbonyl content was found to be at its highest on the 55th day of processing (*p* < 0.05). It is worth noting that the color and flavor scores of the treated samples were markedly improved in comparison to the control samples. Moreover, the TBARS and carbonyl group contents were drastically reduced relative to the control samples (*p* < 0.05). This evidence suggests that nitrite supplementation can effectively delay color and flavor deterioration while suppressing lipid and protein oxidation during sausage processing.

### 3.2. Effect of Nitrite Addition on Peptide Concentration in Sausage Processing

**Figure 2:**
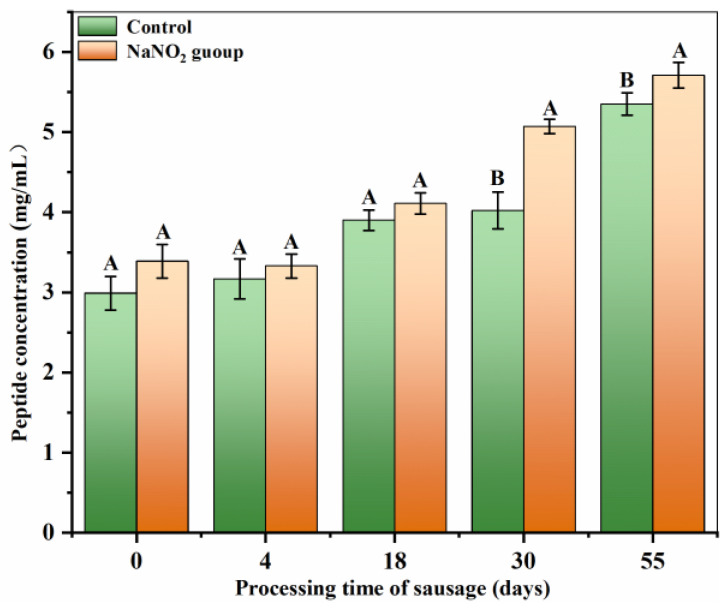
Changes in peptide concentration during sausage processing. Different uppercase letters in a column indicate significant differences among treatments during storage time (days) for each treatment (*p* < 0.05).

During the sausage fermentation process, proteins such as sarcoplasmic and myofibrillar proteins are gradually degraded to form small molecular substances, such as polypeptides. As depicted in Figure 2, the polypeptide concentration of both groups underwent an increase with the increasing in processing time. Moreover, the treated samples exhibited notably enhanced polypeptide concentrations in comparison to the control samples (*p* < 0.05), thereby indicating that the protein degradation was augmented with increasing sausage fermentation time. In addition, nitrate supplementation markedly increased the polypeptide content in fermented sausages. The sausage peptide concentration is generally influenced by several factors, including raw material, type and activity of muscle enzymes, processing conditions, and processing time [20,26]. The peptide concentration of the treated samples was considerably higher compared to the controls, presumably because of nitrite supplementation, which modulates peptide enzymatic activity. Several reports have revealed that the peptidase activity declines due to the gradual decrease in water content within the sausage during fermentation [27]. Moreover, Ma et al. reported that nitrite is decomposed into iron-bound NO during fermentation, which stabilizes the peptidase structure and delays the decline in peptidase activity, thereby resulting in enhanced peptide concentrations in the treated versus control samples [28].

### 3.3. Effect of Nitrite Addition on Antioxidative Capacity

In vitro antioxidant activity assay is a robust assessment tool for polypeptide antioxidant status evaluation. Despite the presence of multiple evaluation methods for the assessment of in vitro polypeptide antioxidant activity, no single procedure to date has measured net polypeptide antioxidant activity. Thus, herein, we selected four distinct antioxidant measurement procedures for the evaluation of the antioxidant potential of polypeptides extracted from sausage at various stages of fermentation.

**Figure 3:**
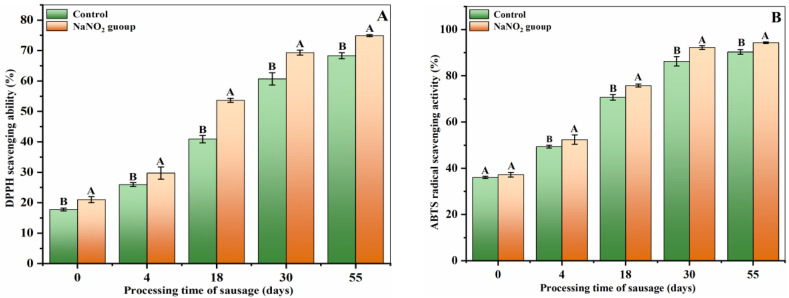

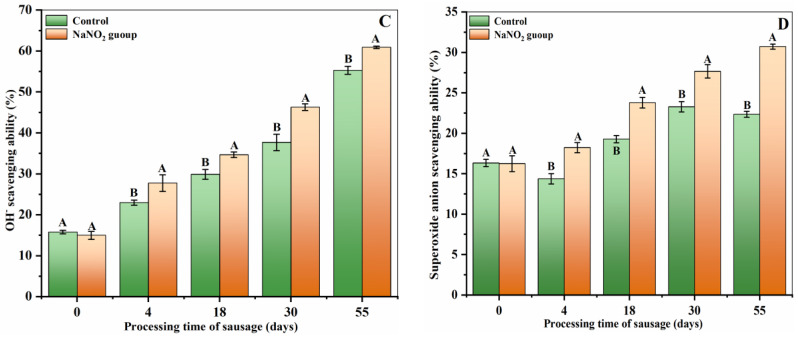
Changes in the antioxidant capacity: (**A**) DPPH scavenging ability, (**B**) ABTS scavenging ability, (**C**) hydroxyl radical scavenging ability, (**D**) superoxide anion free radical scavenging ability) of polypeptides during sausage processing. Different uppercase letters in a column indicate significant differences among various treatments during storage time (days) for each treatment (*p* < 0.05).

DPPH is a fat-soluble free radical [29]. In the presence of a hydrogen-donating antioxidant, its lone electron pair is typically paired, the absorbance decreases within a certain wavelength range, and the attenuation degree is quantitatively associated with the number of absorbed electrons [30]. Hence, DPPH scavenging ability is frequently employed to assess the free radical scavenging ability of antioxidants. The polypeptide DPPH FRSA of the treated and control samples is presented in Figure 3A. Based on our analysis, the DPPH scavenging of all samples increased with the increase in processing duration over the 55-day observation period. This may be due to a reduction in the molecular weight of the peptide [31]. Meanwhile, a similar phenomenon was observed in Iberian dry-fermented sausages [32], Spanish dry-cured ham [33], and Chinese Xuanwei ham [34]. Throughout the observed period, the capacity to scavenge DPPH free radicals was substantially enhanced in the treated samples in comparison to the control samples (*p* < 0.05). This evidence suggests that nitrite supplementation strongly enhances the antioxidant capacity of polypeptides in sausages.

The ABTS free radical assay is a reliable and frequently used procedure for quantifying antioxidant activity. In this method, free radicals are quenched to form ABTS free radical complexes [35]. The antioxidant ability of ABTS free radicals within the control and treated samples is presented in Figure 3B. With the increment in processing durations, the ABTS FRSA increased. The control sample FRSAs were 36.07 ± 0.45% (0 d), 49.37 ± 0.63% (4 d), 70.70 ± 1.21% (18 d), 86.27 ± 2.02% (30 d), and 90.35 ± 0.98 (55 d), respectively. In contrast, the treated sample exhibited FRSAs of 37.23 ± 0.98% (0 d), 52.43 ± 2.03% (4 d), 75.78 ± 0.66% (18 d), 92.26 ± 0.83% (30 d), and 94.34 ± 0.32 (55 d), respectively. Generally, samples with reduced molecular weights exhibited more ABTS FRSA compared to the samples with increased molecular weight. This may explain the difference in data between the treated and control samples [36].

The hydroxyl radical is currently known as the most harmful free radical for living organisms. It causes extensive oxidative damage to biological macromolecules, namely carbohydrates, proteins, nucleic acids, and lipids [37]. Therefore, it is critical to assess antioxidant molecule activities to neutralize this reactive oxygen species. Based on our hydroxyl radical assay, the treated sample polypeptides exhibited a markedly enhanced scavenging ability relative to the control sample polypeptides. Although the hydroxyl radical assay does not directly induce oxidation, it can generate an active hydroxyl radical assay when catalyzed by metal ions. Moreover, excess $O_{2}^{.-}$ can directly cause oxidative damage to tissue [38]. The results of the $\;O_{2}^{.-}$ assay revealed that the nitrite supplementation significantly enhanced polypeptide activities in the sausage (Figure 3C). This phenomenon may be due to the presence of a greater number of short-chain peptides in the treated samples, as several prior studies have demonstrated an association between the peptide chain length and the hydroxyl FRSA of peptides [37,39].

### 3.4. Influence of Nitrite Supplementation on Peptide Iron-Chelating Activity during Sausage Processing

**Figure 4:**
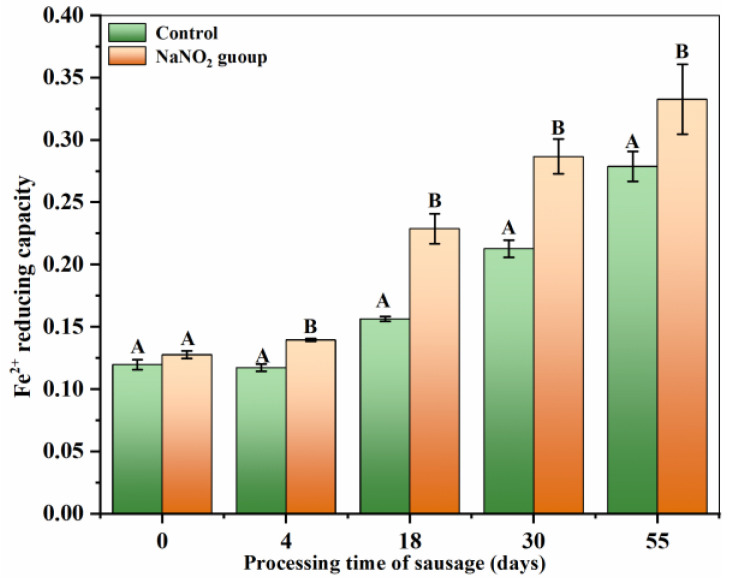
Changes in the iron-chelating activity of polypeptides during sausage processing. Different uppercase letters in the columns indicate significant differences among treatments during storage time (days) for each treatment (*p* < 0.05).

Iron, a trace element, can serve as a peptidase cofactor to modulate the content and activity of polypeptides in sausage [40]. In its free form, ferrous iron (Fe^2+^) generates hydroxyl radicals from hydrogen peroxide and organic hydrogen peroxide, and is, thus, considered a pro-oxidant [41]. Figure 4 illustrates that the Fe^2+^ activity underwent a gradual increase, which may be due to an increase in the extent of protein hydrolysis. This, in turn, exposed and released substances such as aspartic acid, glutamic acid, and histidine, thereby enhancing the chelating capacity with increasing processing time [42]. At any stage of processing, the Fe^2+^ capacity of the treated sample was markedly elevated compared to the controls. This may be responsible for the nitrite-based prevention of the proteolytic breakdown of chelating residues [43].

### 3.5. Mass Spectrometric Determination of Peptide Sequences during Sausage Processing

Polypeptide activity is closely related to its molecular weight and amino acid sequence [35]. Samaranayaka and Li reported that certain hydrophobic amino acids (such as Leu, Val, Met, Phe, Pro, Ala, and Trp) in polypeptides bear critical roles in scavenging free radicals [44]. In the case of Tyr- and Trp-containing polypeptides, the polypeptide’s antioxidant properties are substantially enhanced, since these amino acids are excellent proton donors. Alternatively, His, Asp, and Glu enhance a peptide’s metal-chelating characteristics [25,40]. Herein, we identified 12 polypeptides in the treated and control samples and observed notable differences among them at 0 d (Table 1). The LPGGGHGDL polypeptide is derived from the actin-associated protein 10 and was highly expressed in the treated samples. LPGGGHGDL possesses the most hydroxyl FRSA in Jinhua ham and serves an essential function in hydroxyl FRSA [0]. The presence of LPGGGHGDL may explain the difference in the antioxidant activity between the treated and control samples at 0 d. After 4 days of sausage fermentation, the titin, histone deacetylase-8, and integrin-α-3 proteins were identified [45]. Gallego, Mora, and Toldrá reported that TKYRVP not only possesses anti-inflammatory activity, but also exhibits marked antioxidant activity in dry-cured ham [46]. Moreover, the potential of 1 mg/mL FLKMN in Jinhua ham to scavenge both DPPH and hydroxyl radicals was determined to be 65% and 60%, respectively [47]. Escudero et al. identified SAGNPN in dry-cured ham [45], and its ability to scavenge DPPH radicals at 1.5 mg/mL was measured at 50%. Correspondingly, only one antioxidant peptide, TSNRYHSYPWG, was identified in the control group. TSNRYHSYPWG is derived from a serine/threonine protein kinase and not only possesses antioxidant properties, but also has ACE inhibitory, anti-inflammatory, and other functions [46]. Two novel antioxidant peptides were identified in the treated samples following 18 d of sausage processing, exhibiting significantly elevated reduction capabilities. GLAGA is derived from collagen VII and demonstrates enhanced reducing power in Spanish dry-cured ham, particularly 0.5 AU at 1 mg/mL [45]. Alanine (A) and glycine (G) have been identified multiple times in previous publications. The presence of Leu (L) also contributes to the peptide’s antioxidant activity [48,49]. LPGGGT discovered in Jinhua ham has a DPPH FRSA of approximately 65%, a Fe^2+^ chelating ability of 55%, and an inhibitory activity of approximately 45% at 1.0 mg/mL [26]. Another antioxidant polypeptide, AEEEYPDL, was discovered after 30 days of sausage processing. Mora, Escudero, Fraser, Aristoy, and Toldrá reported that the DPPH FRSA of AEEEYPDL was 95.7 % ± 0.3 at 3 mg/mL, and it possessed an excellent reducing capability [40]. With the extension of the sausage processing time, many antioxidant peptides reported in previous studies were found in the treated and control samples. SNAAC, HCNKKYRSEM, and TSNRYHSYPWG were identified in the Spanish dry-cured ham and were shown to possess significant antioxidant capacity [46]. During the entirety of the sausage processing, various types of known antioxidant polypeptides were markedly elevated in the treated samples as opposed to the control.

## 4. Conclusions

Our analyses revealed that nitrite supplementation strongly enhanced polypeptide contents in fermented sausages in comparison to controls. Moreover, the FRSA of DPPH radicals, ABTS radicals, hydroxyl radicals, superoxide anions, and Fe^2+^ chelation activity were also upregulated in the treated samples. Upon further analysis using LC-MS, it was observed that the treated samples manifested a greater variety of antioxidant active polypeptides in comparison to the controls. Given this evidence, it can be concluded that nitrite supplementation positively modulates antioxidant polypeptide formation in fermented sausage, and potentially enhances sausage quality.

## Figures and Tables

**Table 1:** Types of polypeptides of sausage during processing by LC-MS.

Treatment Group		Control Group	
Peptide Sequence		Peptide Sequence	
**Day 0**			
PGPGPAPGPGPAS	Transcription factor MafF	RACCPGWGG	SCO-spondin
AQSVGGGCC	Ras-related protein Rab-21	GPGSGG	Myozenin-1 (Calsarcin-2)
ACPALGTKSC	Reticulon-3	SRVAGVLGF	N-terminal kinase-like protein
GGGGGGGDM	U1 small nuclear ribonucleoprotein 70 kDa	VVGDGAVGKTCLL	Cell division control protein 42 homolog
GPAGDGDAGGR	Envelope glycoprotein B	EGPQGPPGPVG	Collagen alpha-1(XI) chain
LLLLLLP	Protein shisa-5	VGAVLPGPLLQ	Glycerate kinase
KGIGKMGLGALVLT	Genome polyprotein	PGAAGGAEDGFF	Coatomer subunit alpha
AHKILPVLCGLT	N-terminal kinase-like protein	GPGYYNPNGH	O(6)-methylguanine-induced apoptosis 2
SQLSLHLPPR	TLD domain-containing protein 2	KDTPRLSLLLVIL	Melanoma-associated antigen D4
MADPR	Tubulin-specific chaperone A	GGGGGGGDM	U1 small nuclear ribonucleoprotein 70 kDa
AGPEPEPPL	Palmitoyltransferase ZDHHC5	MCGGGLVCC	Claudin-5
LPGGGHGDL	Actin-related protein 10	AHKILPVLCGLT	N-terminal kinase-like protein
**Day 4**			
SGAGGGGGGGGGGGGGGGG	Calpain small subunit 1	PGAAGGAEDGFF	Coatomer subunit alpha
PGPMGPPGLAGP	Collagen alpha-1(I) chain	ALAPGHLGGLVL	Homeobox protein PKNOX1
PGGGGGGAGGRLA	Neurexin-1-beta	KDTPRLSLLLVIL	Melanoma-associated antigen D4
KDTPRLSLLLVIL	Melanoma-associated antigen D4	GGGGGGGDM	U1 small nuclear ribonucleoprotein 70 kDa
GRFKRFRKKFKKLFKKLSP	Cathelicidin-6	LVPPPTLLVP	Sine oculis-binding protein homolog
TKYRVP	Titin	VGDGAVGKTCLL	Cell division control protein 42 homolog
FLKMN	Histone deacetylase 8	TSNRYHSYPWG	Serine/threonine protein kinase
SAGNPN	Integrin α-3	PTGAPPGGGAL	D site-binding protein
EEPSSCSAMAMGR	DNA-(apurinic or apyrimidinic site) lyase 2	VGAVLPGPLLQ	Glycerate kinase
PPHGEAKAGSSTLPP	Brefeldin A-inhibited guanine nucleotide-exchange protein 1	KDTPRLSLLLVIL	Melanoma-associated antigen D4
VNGFGR	Glyceraldehyde-3-phosphate dehydrogenase		
EEPSSCSAMAMGR	DNA-(apurinic or apyrimidinic site) lyase 2		
**Day 18**			
GKVEADVAGH	Myoglobin	DVIQTGVDNPGHPF	Creatine kinase M-type
PFGNTHNKY	Creatine kinase m-chain	LGVTKDAGDEDL	DnaJ homolog subfamily B member 14
DVGDWRKNV	Troponin-I	GTDSALHRIMEVIDAITTT	6-phosphofructo-kinase
VHIITHGEEK	Myosin light chain 2	ASHHDINDASRGTLSS	Poly(A) RNA polymerase GLD2
HAKHPSDFGA	Myoglobin	DSVNAQADRAF	Testis-specific Y-en-coded-like protein 1
VGGRWK	Troponin-T	PQGALSLEADGHPAAR	Uncharacterized protein KIAA1462 homolog
FAGDDAPRAVFPS	Actin	AGPNSPTGGGGGGGSGGTR	Zinc finger SWIM domain-containing protein KIAA0913
EAAPYLRKSEKERIEAQN	Myosin-1	KPVSPLLL	Creatine kinase M-type
GLAGA	Collagen VII	MCGGGLVCC	Claudin-5
LPGGGHGDL	Actin-related protein 10	AHKILPVLCGLT	N-terminal kinase-like protein
LPGGGT	LIM domain-containing protein 1	LPGGGHGDL	Actin-related protein 10
SAGNPN	Integrin α-3	LPGGGT	LIM domain-containing protein 1
TKYRVP	Titin	SAGNPN	Integrin α-3
**Day 30**			
IPGAPGAIPGIG	Elastin	GVKPAKPGVGGLVGPG	Elastin
APGTAGLP	Collagen alpha-1(I) chain	KGIGKMGLGALVLT	Genome polyprotein
PEGGCCN	ETS translocation variant 1	RACCPGWGG	SCO-spondin
LVDGGGPCGGRV	Antigen WC1.1	AEEEYPDL	Creatine kinase
AAPGGKSLALLQCAYP	Putative methyltransferase NSUN3	SNAAC	Myosin heavy chain
GKFNV	Reticulon-3	TKYRVP	Titin
GLAGA	Collagen VII	MDPKYR	Titin
LPGGGHGDL	Actin-related protein 10	HCNKKYRSEM	Dynein heavy chain
LPGGGT	LIM domain-containing protein 1	GGGGGGGGGGGSSLRMSSN	Calcium-activated potassium channel subunit alpha-1
SAGNPN	Integrin α-3	SEPGCP	Mitogen-activated protein kinase 7
AEEEYPDL	Creatine kinase		
VIGGLLLVVALGPG	Surfeit locus protein 4		
IIFLLVIGTLL	Transmembrane protein 245		
**Day 55**			
DLEE	Protein CNPPD1	LPGGGHGDL	Actin-related protein 10
FLKMN	Histone deacetylase 8	LPGGGT	LIM domain-containing protein 1
GKFNV	Reticulon-3	SAGNPN	Integrin α-3
GLAGA	Collagen VII	AEEEYPDL	Creatine kinase
		SNAAC	Myosin heavy chain
LPGGGT	LIM domain-containing protein 1	TKYRVP	Titin
SAGNPN	Integrin α-3	PGGGGGGAGGRLA	Neurexin-1-beta
AEEEYPDL	Creatine kinase	KDTPRLSLLLVIL	Melanoma-associated antigen D4
SNAAC	Myosin heavy chain	ENPFAC	Zinc finger protein OZF
HCNKKYRSEM	Dynein heavy chain	EEPSSCSAMAMGR	DNA-(apurinic or apyrimidinic site) lyase 2
TSNRYHSYPWG	Serine/threonine-protein kinase	KLLSLGKHGRL	Telomerase reverse transcriptase
DPPFQIT	Prolyl endopeptidase FAP	VVGDGAVGKTCLL	Cell division control protein 42 homolog
APPPPAEVP	Troponin T, fast skeletal muscle	PTGAPPGGGAL	D site-binding protein
QPPLLL	Cyclin-dependent kinase 13	PGLIGARGPPGP	Collagen alpha-1(III) chain
		KDTPRLSLLLVIL	Melanoma-associated antigen D4

## Data Availability

The data presented in this study are available upon request from the corresponding author.
